# Molecular analysis of lipid uptake- and necroptosis-associated factor expression in vitrified-warmed mouse oocytes

**DOI:** 10.1186/s12958-020-00588-x

**Published:** 2020-05-04

**Authors:** Da-Eun Um, Hyejin Shin, Dayoung Park, Jeong Min Ahn, Jayeon Kim, Haengseok Song, Hyunjung Jade Lim

**Affiliations:** 1 Department of Biomedical Science & Technology, Institute of Biomedical Science & Technology, Konkuk University, Seoul, South Korea; 2 Present Address: Maria Fertility Hospital, 20 Cheonho-daero, Dongdaemon-gu, Seoul, 02586 South Korea; 3 Department of Veterinary Medicine, School of Veterinary Medicine, Konkuk University, 120 Neungdong-ro, Gwangjin-gu, Seoul, 05029 South Korea; 4 Department of Obstetrics and Gynecology, CHA Fertility Center at Seoul Station, CHA University, Seoul, South Korea; 5 Department of Biomedical Science, College of Life Science, CHA University, 335 Pangyo-ro, Bundang-gu, Sungnam, Gyeonggi-do 13884 South Korea

**Keywords:** Mouse, Oocyte, Vitrification, CD36, RIPK1, Necrostatin-1

## Abstract

**Background:**

We had previously demonstrated that vitrification reduces the levels of certain phospholipid classes, and that oocytes from aged mice show a similar lipidome alteration, even without vitrification. In the current investigation, we examined if vitrification-warming of mouse oocytes from young and aged mice causes any changes in molecular aspects of lipid-associated features.

**Methods:**

Metaphase II (MII) stage oocytes were harvested from young (10–14-week-old) and aged (45–54-week-old) mice by a superovulation regime with PMSG followed by hCG. We examined the status of the intracellular lipid pool and the integrity of the plasma membrane by staining oocytes with BODIPY 500/510 and CellMask live dyes. Expression of lipid uptake- and necroptosis-associated genes was assessed by quantitative PCR analyses, in oocytes from young and old mice, before and after vitrification. Localization patterns of two crucial necroptosis proteins, phosphorylated MLKL (pMLKL) and phosphorylated RIPK1 (pRIPK1) were examined in mouse oocytes by immunofluorescence staining. Necrostain-1 (Nec1), an inhibitor of RIPK1, was used to examine if RIPK1 activity is required to maintain oocyte quality during vitrification.

**Results:**

We confirmed that vitrified-warmed oocytes from aged mice showed noticeable decrease in both CellMask and BODIPY 500/510 dyes. Among the lipid uptake-associated genes, *Cd36* expression was higher in oocytes from aged mice. Necroptosis is a type of programmed cell death that involves damage to the plasma membrane, eventually resulting in cell rupture. The expression of necroptosis-associated genes did not significantly differ among groups. We observed that localization patterns of pMLKL and pRIPK1 were unique in mouse oocytes, showing association with microtubule organizing centers (MTOCs) and spindle poles. pMLKL was also localized on kinetochores of MII chromosomes. Oocytes treated with Nec1 during vitrification showed a decreased survival rate, indicating the importance of RIPK1 activity in oocyte vitrification.

**Conclusions:**

We report that oocytes from aged mice show differential expression of CD36, which suggests that CD36-mediated lipid uptake may be influenced by age. We also show for the first time that pMLKL and pRIPK1 exhibit unique localization pattern in mouse oocytes and this may suggest role(s) for these factors in non-necroptosis-associated cellular processes.

## Background

The cryopreservation of oocytes and embryos is an essential part of assisted reproductive technologies (ART). Cryopreservation methods use various cryoprotectants, such as ethylene glycol (EG) and dimethyl sulfoxide (DMSO), to minimize cellular damage. Vitrification, the currently preferred cryopreservation method [1], utilizes an ultra-rapid freezing procedure using high concentrations of cryoprotectants. With this method, the cytosol and extracellular environment rapidly transform into a glass-like structure without the formation of ice crystals [2].

During vitrification, oocytes are exposed to chemical and physical stressors. Cryoprotectants such as EG, DMSO, and 1,2-propanediol (PROH) affect various aspects of oocyte biology [3, 4]. Physically, oocytes undergo sudden temperature changes from 37 °C to − 196 °C within a second. These chilling injuries attack the plasma membrane, which is comprised of phospholipids, cholesterol, and other lipid compounds [5–7]. These lipids are the frontal barrier of the cell and serve to protect the intracellular structures [8, 9]. The composition of membrane lipids is a crucial factor that determines membrane fluidity and affects the sensitivity to freezing-associated cellular damage [6]. We previously demonstrated that oocytes undergoing vitrification and warming show a decrease in the levels of multiple phospholipid classes [7]. Moreover, oocytes from aged mice show lipidome alterations, with significant reduction in the levels of several groups of phospholipids, which are the main component of the cell membrane [10]. Thus, it is plausible to hypothesize that during vitrification, oocytes from aged individuals are more vulnerable to phospholipid damage than the oocytes from young individuals.

Scavenger receptor class B type1 (SRB1), scavenger receptor class B type2 (SRB2), and cluster of differentiation 36 (CD36) are integral membrane proteins belonging to the class B scavenger receptor family [11]. These proteins take up phospholipids, high-density lipoproteins (HDL), and low-density lipoproteins (LDL) into the cell as a source of fatty acids [11]. Fatty acids taken up from the extracellular environment create a vital lipid pool that is used for cellular lipid metabolism [12]. Appropriate levels of cholesterol are used for the production or storage of energy sources and the synthesis of phospholipids [13]. Excess cholesterol in mouse oocytes is associated with premature egg activation and compromised fertility [14].

With respect to the integrity of membranous subcellular structures and the plasma membrane, members of the endosomal sorting complex required for transport (ESCRT) are crucial components [15]. ESCRT factors are involved in a wide range of membrane-associated events, such as endosomal maturation, membrane repair, membrane neck-severing during cytokinesis, and exosome formation [15, 16]. Recently, certain ESCRT factors, such as charged multivesicular body protein 4B (CHMP4B) and tumor susceptibility gene 101 (Tsg101), were shown to be involved in minimizing the damage to the plasma membrane during necroptotic cell death [17]. Necroptosis is a form of programmed cell death [18] that is mediated by receptor-interacting kinase 1 (RIPK1), receptor-interacting kinase 3 (RIPK3), and mixed lineage kinase-like (MLKL). The last effector of necroptosis, the phosphorylated MLKL (pMLKL), induces phosphatidylserine (PS) exposure along with small membrane bleb formations [17, 19, 20]. The damaged membrane eventually loses its integrity and becomes permeable, resulting in cell rupture [21, 22].

In this study, we examined if the expression of aforementioned genes associated with membrane integrity is influenced by age or vitrification process in mice. Herein, we focused on two groups of genes involved in two aspects of membrane biology, i.e., lipid uptake and necroptosis.

## Materials and methods

### Mice

Mice were housed in constant temperature at 23 °C ± 1 °C with 40% humidity under a 12 h light–dark cycle. Eight-week-old female B6D2F1 [BDF1, cross between C57BL/6 J (B6) female x DBA/2 J (D2) male] mice were purchased from Orient Bio (Gyunggi-do, Korea). Mice between 10 and 14-weeks-old were used in the “young” group, whereas mice older than 45 weeks were used in the “old” group. All experiments were conducted in accordance with the policies of the Konkuk University Institutional Animal Care and Use Committee (Approval number KU17067). Mice were sacrificed under anesthesia, and all efforts were made to minimize suffering of the mice.

### Oocyte collection

Mice received 10 IU of pregnant mare’s serum gonadotropin (PMSG, Sigma-Aldrich, St. Louis, USA) intraperitoneally, and 10 IU of human chorionic gonadotropin (hCG, Sigma-Aldrich) 48 h later (7–8 pm). Ovulated cumulus-oocyte complex (COCs) were retrieved from the oviduct 13 h post-hCG (8–9 am). 20 IU of PMSG and hCG injections were used for better induction in older mice, as in previously conducted research [10, 23]. To remove the cumulus cells, the COCs were treated with hyaluronidase (300 mg/ml, H4272, Sigma Aldrich) in Quinn’s Advantage Medium with HEPES (SAGE Media, ART-1023, Trumbull, CT, USA) for 2 min at room temperature. Denuded metaphase II (MII) oocytes were washed and collected in Quinn’s Advantage Medium with HEPES containing 20% fetal bovine serum (FBS, Gibco; Grand Island, NY, USA).

### Vitrification and warming

Vitrification was performed as previously described by Cha et al. [24]. Ethylene glycol (EG, 102466, Sigma-Aldrich) and dimethyl sulfoxide (DMSO, D2650, Sigma-Aldrich) were used as cryoprotectants in the vitrification solution. Oocytes were equilibrated in PBS based media containing 7.5% EG, 7.5% DMSO, and 20% FBS for 2.5 min, and then transferred to media containing 15% EG, 15% DMSO, and 0.5 M sucrose (Fisher Scientific, Fair Lawn, USA) for 20 s. Equilibrated oocytes (20 to 25) were loaded onto a copper grid (Ted Pella Inc., Redding, USA) and dipped directly into liquid nitrogen (LN_2_). Vitrified oocytes were stored in a LN_2_ tank for 2–4 weeks. For the warming procedure, the grid was taken out from the LN_2_ tank and serially incubated in 20% FBS containing PBS with descending concentrations of sucrose (0.5, 0.25, 0.125, 0 M) for 2.5 min each. The vitrified-warmed oocytes were washed in Quinn’s Advantage medium containing HEPES and 20% FBS. Washed oocytes were cultured in M16 media (M7292, Sigma-Aldrich) at 37 °C, in 5% CO_2_ for 1 or 3 h. For Necrostatin-1 (Nec1, N9037, Sigma-Aldrich) supplementation, 1 μM of Nec1 [25] was added to the final vitrification solution (15% EG, 15% DMSO, and 0.5 M sucrose). Oocytes without any marked morphological deformation and discoloration under an inverted microscope were considered as survived ones and used for further analysis.

### RNA extraction and quantitative real-time polymerase chain reaction analysis

MII oocytes obtained from multiple mice were pooled and randomly grouped in 20 for RNA extraction. mRNA was extracted from 20 oocytes in all experimental groups, using Dynabeads™ mRNA DIRECT™ Purification Kit (Life Technologies, Forster City, CA, USA) according to the manufacturer’s protocol. The mRNA was kept in − 80 °C before use. First strand cDNA was synthesized from total mRNA sample using a Superscript TM III Reverse Transcriptase (Invitrogen, 18,080–044, Carlsbad, CA, USA), RNaseOUT™ Recombinant Ribonuclease Inhibitor (Invitrogen, 10,777–019), Oligo (dT)_20_, and random hexamer primers (Roche, Basel, Switzerland). Real-time quantitative PCR (qPCR) was performed using 2 μl of oocyte cDNA (equivalent to one oocyte per reaction) and Applied Biosystems™ Power Up™ SYBR™ Green Master Mix (Invitrogen, A25742, Carlsbad, CA, USA) in a final volume of 20 μl on the ABI 7500 real-time PCR system. The PCR conditions were as follows: hold for 10 min at 95 °C, followed by each cycle consisting of denaturation at 95 °C for 15 s, annealing and elongation at 58 °C for 1 min each. The relative gene expression was normalized with *H2afz* mRNA expression and relative quantification was performed using the ddCt method [26, 27]. PCR was performed by using Econo Taq PLUS GREEN 2X Master Mix (Lucigen, Middleton, WI, USA). Three biological replicates were used per experimental group and all reactions were run in duplicates. Primers used are shown in Table 1.

**Table 1:** Primers used in this study

Gene	Sequence (5′-3′)	Product size (bp)	GenBank accession no.	Use
*Srb1*	F: TCT GGC GCT TTT TCT ATC GT R: ACG GCC CAT ACC TCT AGC TT	123	NM_016741.2	qPCR
*Srb2*	F: AGC CGA CGA GAA GTT CGT TT R: CCC GTT TCA ACA AAG TCA TCC A	165	NM_007644.4	qPCR
*Cd36*	F: TCA TGC CAG TCG GAG ATG R: TGG TGC CTG TTT TAA CCC AGT T	102	NM_001159558.1	qPCR
*Tsg101*	F: ATG GCG GTG TCC GAG AGT CAG R: TTG ACA GTT TGA CGG ACG GT	80	NM_021884.3	qPCR
*Ripk1*	F: GAA GAC AGA CCT AGA CAG CGG R: CCA GTA GCT TCA CCA CTC GAC	182	NM_009068.3	qPCR
*Ripk3*	F: CAC ATA CTT TAC CCT TCA GA R: TCA GAA CAG TTG TTG AAG AC	172	NM_019955.2	RT-PCR
*Chmp4b*	F: GGA GAA GAG TTC GAC GAG GAT R: TGG TAG AGG GAC TGT TTC GGG	111	NM_029362.3	qPCR
*H2afz*	F: ACA GCG CAG CCA TCC TGG AGT A R: TTC CCG ATC AGC GAT TTG TGG A	202	NM_016750.3	qPCR
*Mlkl*	F: GAC CAA ACT GAA GAC AAG TA R: CTC ACT ATT CCA ACA CTT TC	114	NM_001310613.1	RT-PCR

### Cell culture and necroptosis induction

L929 fibroblast cell line derived from mouse adipose tissue was obtained from Korean Cell Line Bank (Seoul, Korea). L929 cells were cultured in RPMI1640 media supplemented with *L*-glutamine (300 mg/L), 25 mM HEPES, 25 mM NaHCO_3_, and 10% FBS. To induce necroptosis, cells were treated with a mixture of 30 ng/mL TNFα (PeproTech, Rocky Hill, NJ), 10 μM LCL-161 (R&D Systems, Minneapolis, MN, USA), and 20 μM Z-VAD-FMK (R&D Systems) for 40 min [28]. Cells were fixed and subjected to immunofluorescence staining with anti-pMLKL and anti-pRIPK1 antibodies to establish specificity of these antibodies.

### Immunofluorescence staining

Oocytes were fixed with 4% paraformaldehyde containing 0.05% polyvinyl alcohol in phosphate buffered saline (PFA-PVA) for 10 min. Fixed oocytes were washed three times with phosphate-buffered saline containing 0.05% polyvinyl alcohol (PBS-PVA) for 10 min each. For permeabilization, oocytes were transferred to a solution containing 0.25% Triton X-100 and incubated for 10 min. To prevent nonspecific binding, oocytes were blocked with 2% BSA in PBS for 1 h, followed by incubation with primary antibody at 4 °C overnight. The primary antibodies used were anti-pMLKL (1:100, ab196436, Abcam) [21], anti-pericentrin (1:500, 611,814, BD Bioscience, San Jose, CA, USA) [29], and anti-pRIPK1 (1:150, 31,122, Cell Signaling Technology, Danvers, MA, USA). Following incubation with primary antibodies, oocytes were washed three times with PVA-PBS for 10 min. The oocytes were then incubated with Alexa Fluor 488 or Alexa Fluor 568 conjugated secondary antibody (1:250, Invitrogen) for 1 h at room temperature. DNA was stained with TOPRO-3-iodide (1:250, Invitrogen). The oocytes were mounted on glass slides with Vectashield mounting medium (Vector Laboratories, Peterborough, UK) and observed under the confocal microscope (Zeiss LSM710, Carl Zeiss AG, Oberkochen, Germany). Specificity of anti-pMLKL and anti-pRIPK1 antibodies was confirmed in necroptosis-induced L929 cells [21]. In all experiments, rabbit IgG was used as a negative control and it did not generate any specific signal.

### Live imaging of oocytes by confocal microscopy

Oocytes were washed with M16 media three times and stained with CellMask™ Plasma Membrane Stain (2.5 μg/ml; C10046, Life technologies), BODIPY 500/510 dodecanoic acid (10 μg/ml; D-3823, Invitrogen), or ER Tracker™ Red dye (1 μg/ml; E34250, Invitrogen) for 30 min. The oocytes were rinsed with fresh M16 media three times and transferred to a glass bottom confocal dish. Live images of oocytes were obtained directly with a confocal microscope (Zeiss LSM710).

### Statistical analysis

Data analysis and graph preparation were done using GraphPad Prism 5 software. For statistical analysis, Student’s *t*-test or one-way analysis of variance (ANOVA) were conducted on the experimental groups. Tukey’s range test was then performed to identify whether a significant difference exists among the groups. Statistical significance was depicted as *: *p* < 0.05, **: *p* < 0.01 and ***: *p* < 0.001.

## Results

### Ovulation rate decreases in aged BDF1 mice

**Fig. 1:**
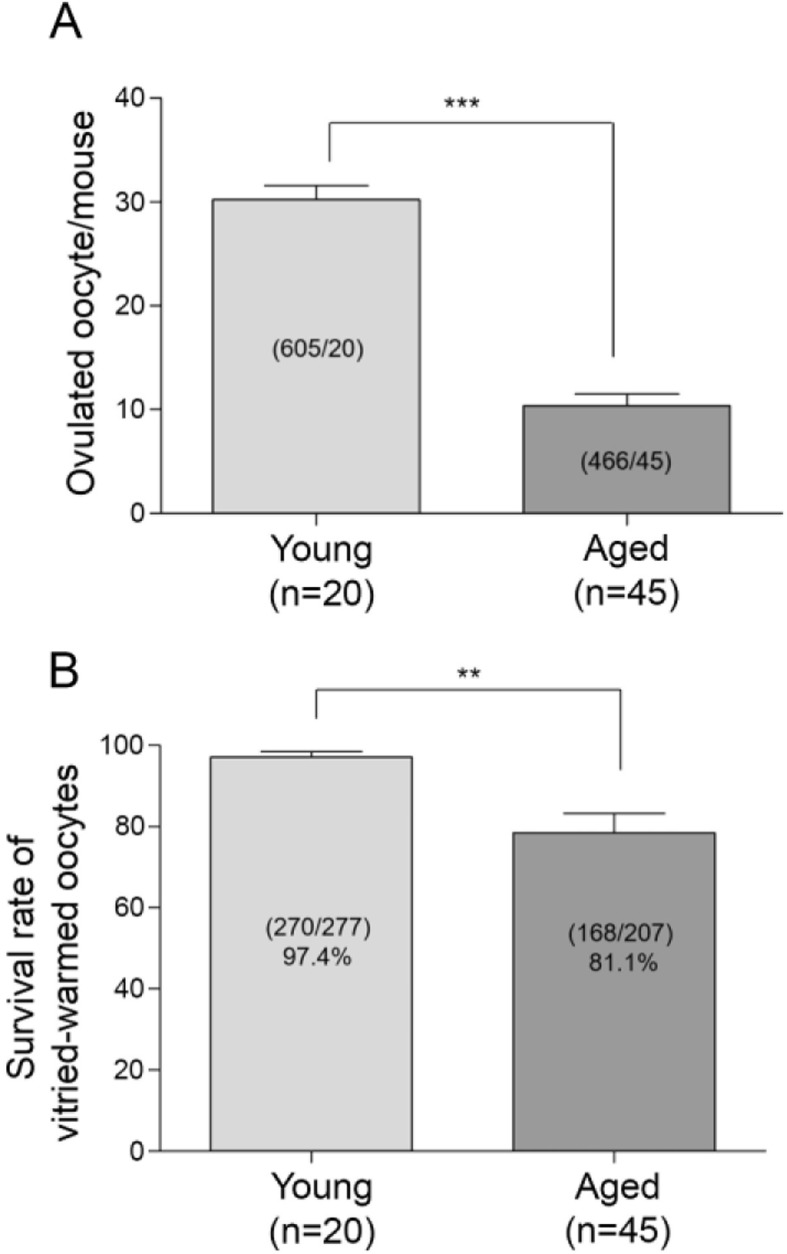
The ovulation and survival rates after vitrification in the oocytes from the young and aged BDF1 mice. **a** Young (10–14 weeks) and aged (45–54 weeks) BDF1 mice were induced to superovulate with PMSG and hCG injections, and MII oocytes were retrieved at 13 h post-hCG. The graph depicts the average number of ovulated oocytes per mouse. A total of 605 MII oocytes were retrieved from 20 young mice and 466 oocytes from 45 aged mice. The value represents mean ± S.E.M. Statistical significance was measured by a Student’s t-test (two-tailed). ***, *p* < 0.0001, t = 10.33, df = 63. **b** Viability of vitrified-warmed oocytes from young and aged BDF1 mice. A portion of MII oocytes from (**a**) was vitrified and stored in LN_2_ for 2 to 4 weeks. The numbers were added from 8 independent experiments. The number of survived oocytes per total number of oocytes is shown within each bar. The value represents mean ± S.E.M. Statistical significance was measured by a Student’s *t*-test (two-tailed). **, *p* = 0.0022, t = 3.743, df = 14

Different strains of mice are commercially available, and they generally show differing degrees of reproductive performance [30]. Herein, we first determined the ovulation efficiency of BDF1 strains. BDF1 mice are a hybrid line generated from crossing a female C57BL/6 to a male DBA/2 [31]. Female mice at 10–14 weeks are considered fully mature with high reproductive performance, and after 45 weeks of age, mice are considered to be at the end of their reproductive lifespan [32]. Mice were induced to superovulate with PMSG and hCG injections, and MII oocytes were retrieved at 13 h post-hCG from the oviducts of young and aged mice. As shown in Fig. 1a, the ovulation rate significantly decreased in aged BDF1 mice. Young mice ovulated 30 ± 1.3 oocytes per mouse (*n* = 20) and the aged group ovulated 10.3 ± 1.1 oocytes (*n* = 45). Young and aged oocytes did not exhibit any morphological difference under a stereomicroscope (data not shown). A portion of oocytes from BDF1 were subjected to vitrification-warming as shown in Fig. 1b. Vitrified-warmed oocytes without noticeable deformities and discoloration were considered survived. While the survival rate of vitrified-warmed oocytes from young mice was 97.4%, that of vitrified-warmed oocytes from aged mice was 81.1%, showing a significant difference between these groups. This result suggests that maternal aging has an adverse effect on the survival of vitrified-warmed oocytes.

### Assessment of lipid-associated cellular structures in vitrified-warmed oocytes from aged mice

**Fig. 2:**
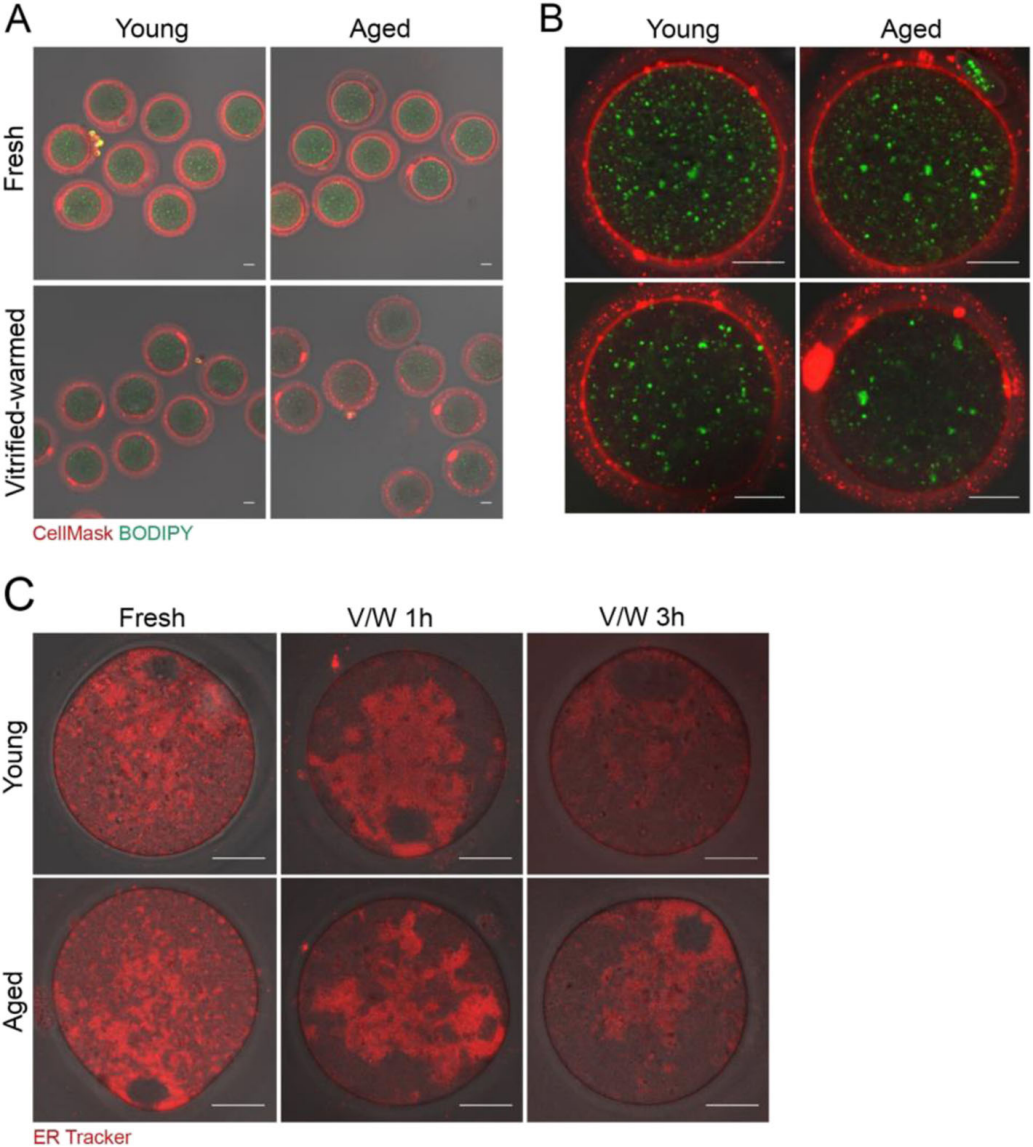
Fluorescence live imaging showing lipid components of mouse oocytes before and after vitrification-warming. Vitrified MII oocytes from young or aged BDF1 mice were stored in LN_2_ for 2 to 4 weeks. Oocytes were warmed and stabilized in M16 media for 1 h. **a-b** Fresh and vitrified-warmed oocytes were stained with CellMask™ Plasma Membrane Stain (2 μg/ml) and BODIPY 500/510 (10 μg/ml) in M16 media. A total of three experiments using randomly picked oocytes were performed, and representative images are shown (oocytes used are: 14 fresh young, 13 fresh aged, 13 vitrified-warmed young, and 14 vitrified-warmed aged). Scale bar represents 20 μm. A set of enlarged images is shown in (**b**). **c** Morphology of endoplasmic reticulum (ER) was examined by fluorescence live imaging using ER Tracker™ Red dye (1 μg/ml). Vitrified-warmed oocytes were processed for staining after 1 or 3 h of stabilization in M16 media. The oocytes numbers are the following: fresh young (9), fresh aged (10), vitrified-warmed young at 1 h (10), 9 vitrified-warmed aged at 1 h (9), vitrified-warmed fresh at 3 h (8), and vitrified-warmed aged at 3 h (8). Two or three experiments were independently performed with randomly picked oocytes from each group. White scale bar represents 20 μm

We had previously used two dyes to monitor the status of the plasma membrane and intracellular lipid content of vitrified-warmed mouse oocytes in real-time [7]. We had used the following: BODIPY fatty acid 500/510, a fluorescent fatty acid analog that labels intracellular fatty acids, and CellMask™ Deep Red that stains the plasma membrane [7]. When examined by confocal microscopy at 1 h of thawing, vitrified-warmed oocytes showed a decrease in the intracellular lipid content and intensity of plasma membrane staining [7]. Oocytes from aged mice exhibited similar changes even without the vitrification-warming process [10]. Thus, we examined if vitrification-warming of oocytes from aged mice showed an aggravated condition of intracellular lipid content and plasma membrane. Fresh and vitrified-warmed oocytes from young and aged mice were stained with CellMask™ Deep Red and BODIPY fatty acid 500/510. Figure 2a shows the overall staining status of fresh and vitrified-warmed oocytes from young and aged mice. All oocytes demonstrated an abundant amount of small lipid droplets throughout the ooplasm. However, vitrified-warmed oocytes showed a decrease in these puncta. As shown in Fig. 2b, the amount of BODIPY 500/510-positive lipid droplets was noticeably lower in vitrified-warmed oocytes from aged mice. CellMask™ Deep Red live imaging revealed uniform pattern of ring-shaped plasma membrane peripheries in fresh young and aged groups, whereas vitrified-warmed oocytes from aged mice showed low intensity and discontinuous staining patterns (Fig. 2b).

The endoplasmic reticulum (ER) is a major endomembranous subcellular organelle in the cell, which is the site of lipid synthesis [33] and is the source of calcium ions released at the time of fertilization in oocytes [34]. To determine whether there is any difference in ER configuration or morphology in aged oocytes, we stained fresh and vitrified-warmed oocytes with ER Tracker Red, as shown in Fig. 2c. The ER patterns in fresh oocytes did not differ between oocytes from different age groups. At 1 h after vitrification-warming, the ER structure did not seem to have recovered fully in perinuclear localization in both young and aged groups. At 3 h, a normal ER pattern was restored in all groups, showing cortical clusters (Fig. 2c). ER cortical clusters are one of the distinguishable phenotypes in MII oocytes preparing for calcium release at impending fertilization [34].

### Expression of lipid uptake-associated genes in vitrified-warmed mouse oocytes

**Fig. 3:**
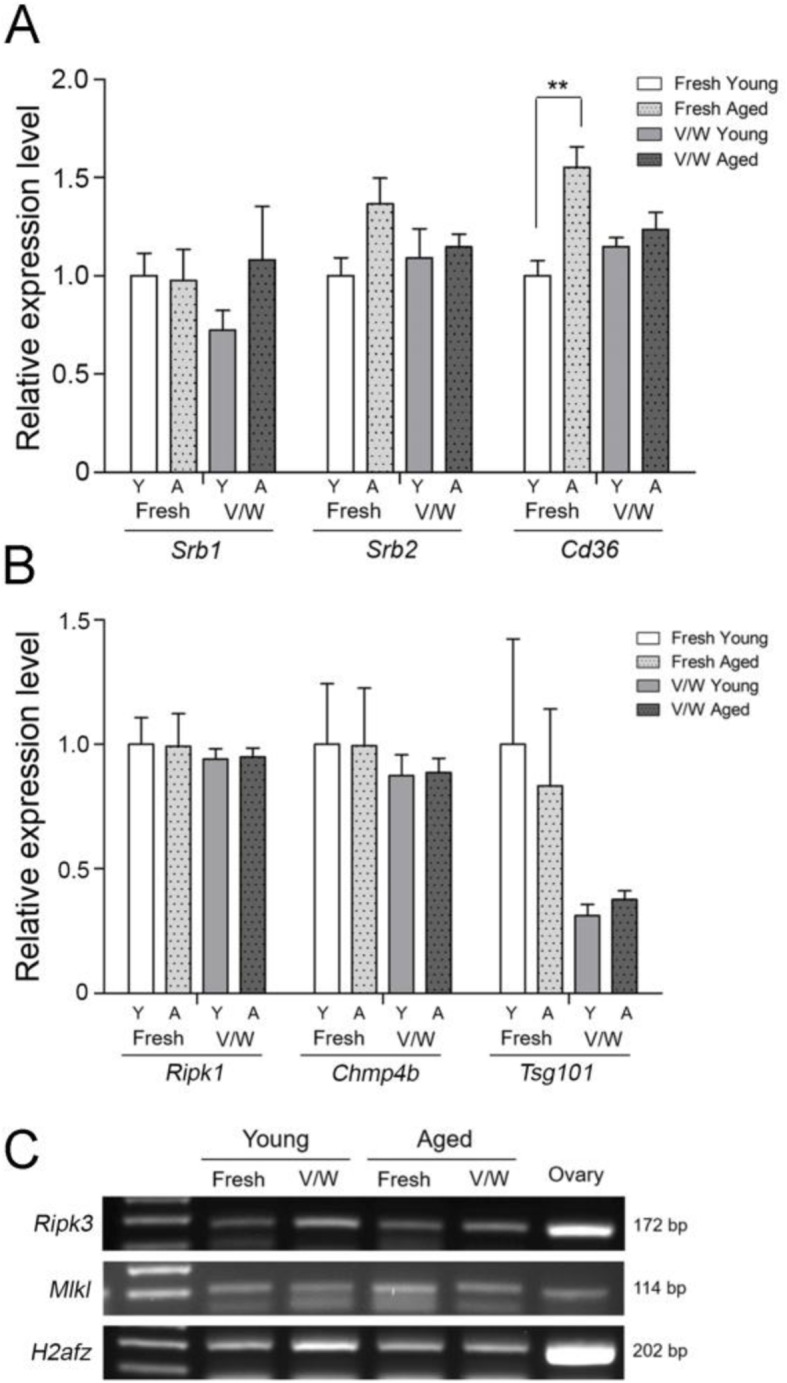
Expression of lipid uptake- and necroptosis-associated genes in mouse oocytes before and after vitrification-warming. MII Oocytes obtained from multiple mice were randomly grouped in 20, and three biological replicates were used for each group. Each sample was run in duplicates for quantitative PCR (qPCR, approximately one oocyte per one reaction). The relative gene expression was normalized with the expression of histone H2A.z (*H2afz*). The statistical analysis was performed with one-way ANOVA and Tukey’s range test. (A) qPCR analyses of *Srb1*, *Srb2,* and *Cd36* expression in fresh and vitrified-warmed (V/W) oocytes were performed for young (Y) and aged (A) mice. **, *p* = 0.0078, F = 8.258, R squared = 0.7559. The values represent the mean ± S.E.M. (B) qPCR of *Tsg101*, *Chmp4b*, and *Ripk1* levels in fresh and vitrified-warmed (V/W) oocytes were performed for young (Y) and aged (A) mice. MII Oocytes obtained from multiple mice were randomly grouped in 20, and three biological replicates were used for each group. No significant difference among groups. (C) *Ripk3* and *Mlkl* were not detectable by qPCR; thus, the presence of their mRNAs was confirmed on gel after RT-PCR

As mentioned, *Srb1*, *Srb2*, and *Cd36* are involved in the uptake of phospholipids, HDL, and LDL into the cell as lipid sources [11]. The expression of these three genes was examined in fresh and vitrified-warmed oocytes of young and aged mice. As shown in Fig. 3a, the expression of *Cd36* was significantly higher in fresh oocytes from aged mice when compared to that in young mice. Other genes did not show significant changes among groups.

### Expression of necroptosis-associated genes in vitrified-warmed mouse oocytes

Next, we examined the expression of major necroptosis effector genes *Ripk1*, *Ripk3,* and *Mlkl* in mouse oocytes. The expression of *Tsg101* and *Chmp4b*, two major counteracting factors of necroptosis, was also examined. Importantly, the expression and role for these genes in mouse oocytes have not been reported. As shown in Fig. 3b, *Ripk3*, *Chmp4b*, and *Tsg101* are expressed in mouse oocytes before and after vitrification-warming, but do not show any significant difference in expression. While the expression levels of *Ripk3* and *Mlkl* were not readable by qPCR analysis, the amplified products after RT-PCR were confirmed on a gel (Fig. 3c). Overall, the data show that effectors and countering factors of necroptosis are present in mouse oocytes without much changes before and after vitrification-warming.

### Unique localization of pMLKL in mouse oocytes

**Fig. 4:**
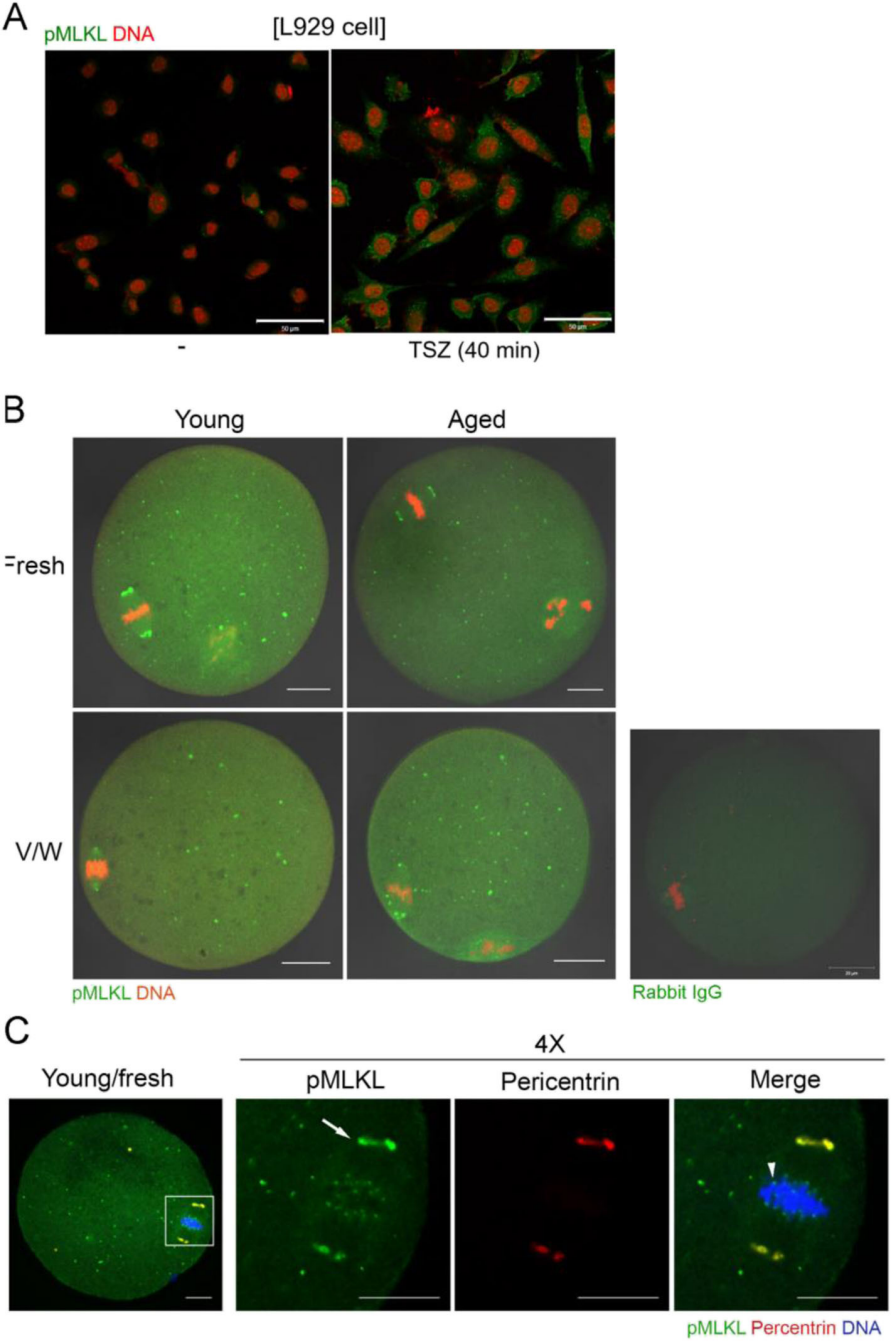
Localization of pMLKL in vitrified-warmed oocytes from young and aged mice. **a** Specificity of anti-pMLKL antibody was confirmed in L929 cells treated with necroptosis-inducing reagents. -, no treatment; TSZ, a mixture of TNFα, LCL161, Z-VAD-FMK. Scale bar represents 50 μm. **b** Immunofluorescence staining of pMLKL in oocytes from young and aged mice before (fresh) and after vitrification-warming (V/W). Oocytes were fixed, permeabilized, and treated with anti-pMLKL antibody at 1:100 (green). DNA was counterstained with TOPRO-3-iodide (1:250). Experiments were repeated three times, using more than 14 oocytes per group. White scale bar represents 20 μm. **c** A representative image showing co-localization of pMLKL and pericentrin. The white boxed area is enlarged four times. The primary antibodies used are, anti-pMLKL (1:100, green) and anti-pericentrin (1:500, red). The arrow indicates the spindle pole and the arrowhead indicates kinetochore. White scale bar represents 10 μm

**Fig. 5:**
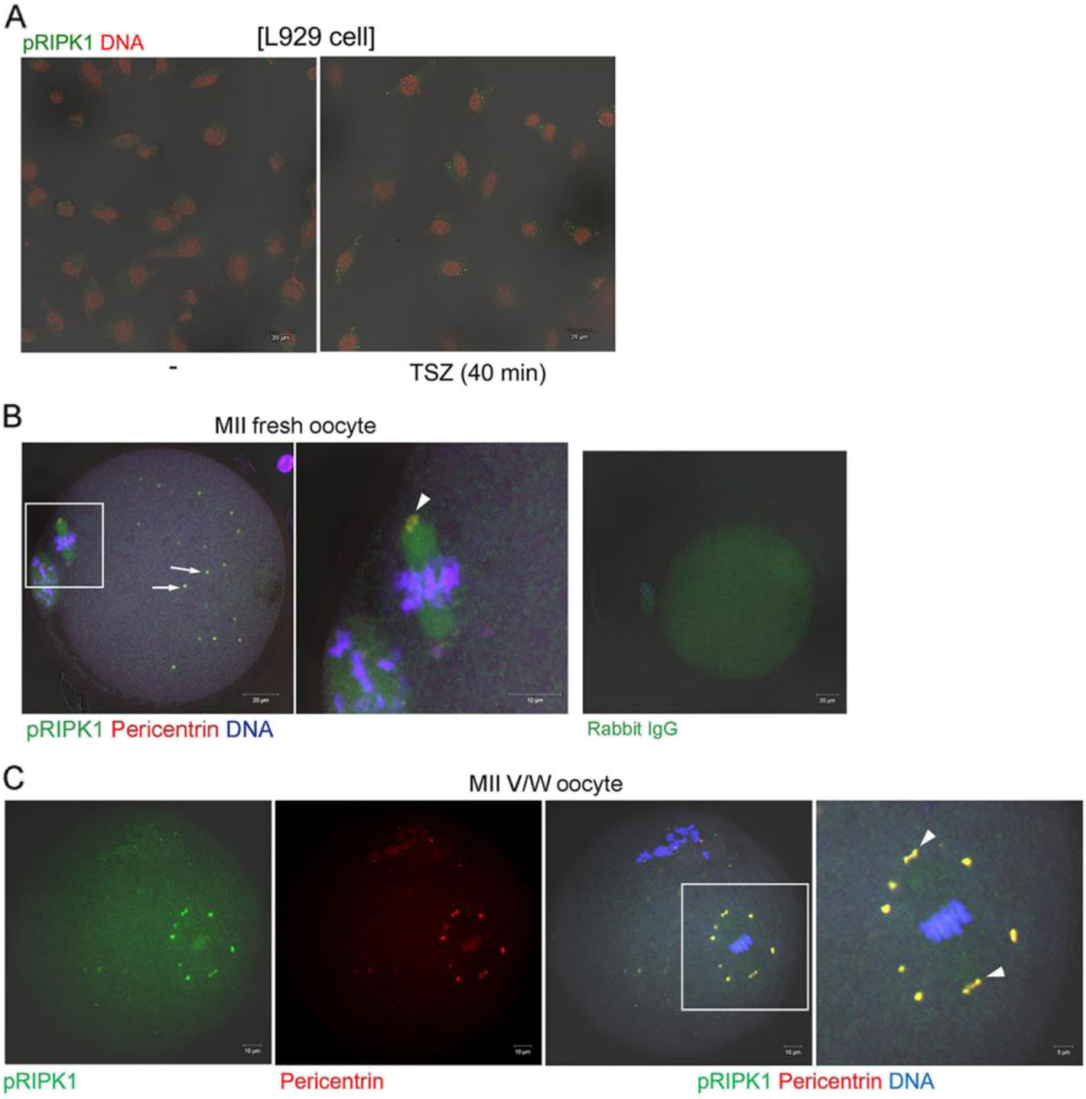
Localization of pRIPK1 in vitrified-warmed oocytes. **a** Specificity of anti-pRIPK1 antibody was confirmed in L929 cells treated with necroptosis-inducing reagents. -, no treatment; TSZ, a mixture of TNFα, LCL161, Z-VAD-FMK. Scale bar represents 20 μm. **b** Immunofluorescence staining of pRIPK1 in MII oocytes from young mice. The primary antibodies used are, anti-pRIPK1 (1:150, green) and anti-pericentrin (1:500, red). DNA was counterstained with TOPRO-3-iodide (1:250). Overlapped signals of pRIPK1 and pericentrin are visualized in yellow. Arrows indicate pericentrin-positive MTOCs and arrowhead indicates the spindle pole. The white boxed area is enlarged on the right. White scale bar represents 20 μm or 10 μm in the enlarged image. **c** A representative image showing co-localization of pRIPK1 and pericentrin. The white boxed area is enlarged four times. The primary antibodies used are, anti-pRIPK1 (1:150, green) and anti-pericentrin (1:500, red). Arrowheads indicate the spindle poles. The white boxed area is enlarged on the right. White scale bar represents 10 μm or 5 μm in the enlarged image

Necroptosis is induced when there are extrinsic stimuli to activate RIPK1, RIPK3, and MLKL, the major effectors of this cell death pathway [35]. The activity of these effectors is regulated by phosphorylation. At the final stage in necroptosis, MLKL proteins are oligomerized and move toward the plasma membrane to induce phosphatidylserine exposure [17]. We investigated subcellular localization of pMLKL and pRIPK1 in mouse oocytes for the first time by using immunofluorescence staining. We first established the specificity of anti-pMLKL antibody in L929 cells treated with a mixture of TNFα, LCL-161, and Z-VAD-FMK (collectively referred to as TSZ) [28]. As shown in Fig. 4a, multiple cytoplasmic puncta of pMLKL appeared in L929 cells treated with TSZ, demonstrating the specificity of this antibody. In mouse oocytes, pMLKL was widely distributed over the ooplasm as small puncta and this pattern was similar in all oocyte groups (Fig. 4b). We observed that pMLKL was also localized in spindle poles in all groups. Therefore, we co-stained oocytes with antibodies to pMLKL and pericentrin, a marker of microtubule organizing center (MTOC) and the spindle pole [29] (Fig. 4c). While some pMLKL-positive puncta were co-localized with pericentrin, suggesting an association with MTOC, pMLKL puncta were more widespread in the cytoplasm than pericentrin. As observed in an enlarged image of the chromosome-spindle complex (Fig. 4c, 4X), pMLKL signals were also clearly visible in kinetochore regions where the spindle meets the chromosomes. To our knowledge, the observed pMLKL pattern in oocytes is unique and has not been reported in other cell systems. Next, we examined the localization of pRIPK1 in young oocytes before and after vitrification (Fig. 5). The specificity of anti-pRIPK1 antibody was also confirmed in TSZ-treated L929 cells (Fig. 5a). Notably, pRIPK1 also showed MTOC-like localization patterns in fresh and vitrified-warmed MII oocytes. MTOC marker pericentrin clearly overlapped with pRIPK1 signals in both groups (Fig. 5b & c). Overall, these observations suggest that pMLKL and pRIPK1 may have a necroptosis-independent role in mouse oocytes.

### Role of RIPK1 evaluated by adding necrostatin-1 during vitrification process

**Fig. 6:**
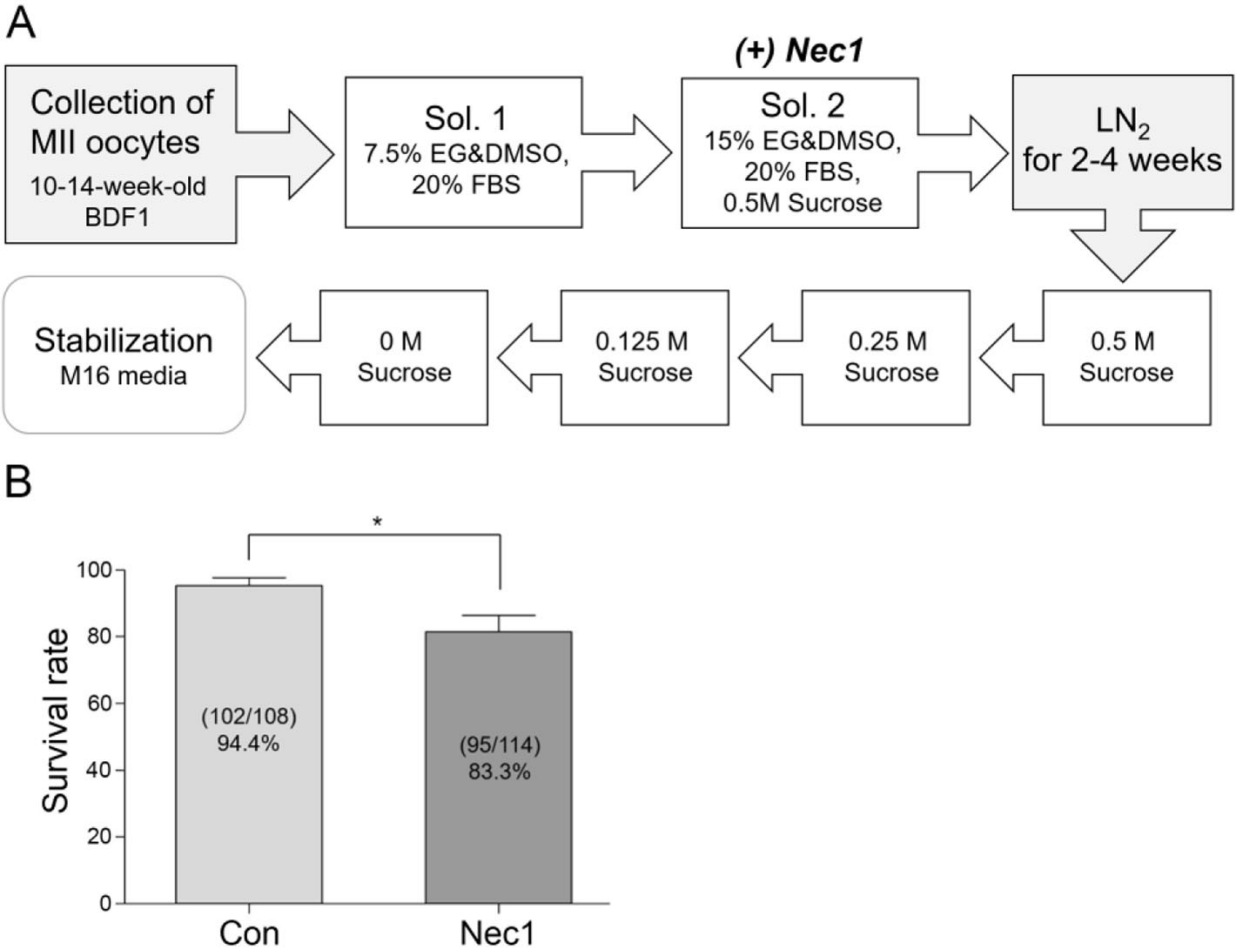
The effect of necrostatin-1 supplementation during vitrification in mouse oocytes. Oocytes from 10 to 14 weeks of age were vitrified and stored in LN_2_ for 2–4 weeks. Necrostain-1 (Nec1, 1 μM) was added to the final vitrification solution (15% EG, 15% DMSO, and 0.5 M sucrose), as shown in (**a**). **b** The experiment was performed 5 times independently using the number of mice given in parentheses. The numbers of survived oocytes per total number of oocytes are shown within each bar. The values represent mean survival rates ± S.E.M. Statistical significance was analyzed by a two-tailed Student’s *t*-test. *, *p* = 0.0346, t = 2.542, df = 8

Necrostatin-1 (Nec1) is a small molecule inhibitor of RIPK1 that is used to block necroptosis in various cell systems [36]. The addition of Nec1 during in vitro maturation was shown to improve survival and developmental competence in mouse oocytes [25]. Oocytes cultured in the presence of 1 μM Nec1 do not show any sign of cytotoxicity (unpublished data, Shin H and Lim HJ). To examine whether RIPK1 activity is important for maintaining oocyte quality during vitrification, Nec1 was added to the final vitrification solution (15% EG, 15% DMSO, and 0.5 M sucrose). After 2–4 weeks in LN_2_, the survival rates of vitrified-warmed oocytes were monitored. As shown in Fig. 6, the control oocytes showed a survival rate of 94.4%, whereas the Nec1-added oocytes showed a survival rate of 81.4%. The result indicates that RIPK1 activity is required for maintenance of optimal oocyte quality during vitrification.

## Discussion

The demands for oocyte cryopreservation have increased in recent years due to several social and health concerns [37, 38]. It is widely known that women older than 35 years of age have a higher chance of harboring chromosomal abnormalities and intracellular organelle dysfunctions [39, 40]. Furthermore, the clinical efficiency of vitrification is significantly lower in oocytes from aged women than those from young women [41]. Women under 35 years of age have high success rates of ART and demonstrate no significant difference with respect to fertilization and implantation rates between the fresh oocyte and vitrificated oocyte. However, in women older than 38 years of age, vitrified-warmed oocytes show low survival, fertilization, and implantation rates [2, 41]. Thus, maternal age is an essential parameter for predicting oocyte quality [42]. Therefore, molecular and cellular changes that might occur in oocytes from older individuals are of important research interest. In the present investigation, we assessed two groups of genes that are associated with lipid biology, i.e. lipid uptake and necroptosis. Vitrification using multiple cryoprotectants and fast cooling inevitably exposed oocytes to chemical and physical stressors. Compared to other cell types, the high surface-to-volume ratio of oocytes may render them more vulnerable to external insults. We previously showed in two independent studies that the vitrification process influences the phospholipid content of mouse oocytes [7], and that the levels of several classes of phospholipids are reduced in oocytes from aged mice [10]. This led us to investigate the expression of lipid uptake-associated genes. Among the three genes we studied (Fig. 3a), the expression of *Cd36* showed a slight increase in fresh oocytes from aged mice. *Cd36* is expressed in both mouse and human oocytes [43], and is implicated in the binding of the sperm to the egg—at the time of fertilization—by recognizing phosphatidylserine on the sperm membrane [44]. In oocytes from aged mice, *Cd36* expression is higher than oocytes from young mice (Fig. 3a). Considering that the levels of certain phospholipids are reduced in oocytes from older mice [10], it may be associated with an increased demand for specific lipid uptake. The significance of increased *Cd36* expression in oocytes from aged mice is currently unknown and requires further investigation.

Necroptosis is one of the programmed cell death pathways that involves the action of RIPK1, RIPK3, and MLKL [35]. The signals for necroptosis include tumor necrosis factor α, FAS ligand, and TNF-related apoptosis-inducing ligand (TRAIL) [18]. With respect to the role of necroptosis in aging, there is evidence that necroptosis is active in the reproductive system of aged male mice [45]. Necroptotic activation was also observed in the epidermal white adipose tissue of aged mice [46]. In the female reproductive organs, necroptosis is suggested to be involved during follicular development in primates [47]. However, to date, there is no available information as to whether the oocytes are subjected to necroptotic activation under specific conditions. Here we showed that components that are crucial for necroptosis are present in mouse oocytes. Vitrification did not influence the expression of these factors significantly. Effectors of necroptosis, including pMLKL and pRIPK1, generally exhibit cytoplasmic puncta patterns that dynamically respond to external stimuli [17, 21]. While pMLKL in oocytes were shown as numerous cytoplasmic puncta, specific signals on spindle poles and kinetochore were observed (Fig. 4). pRIPK1 also showed a unique distribution in oocytes before and after vitrification-warming, on MTOCs. Such unique localization patterns suggest that these factors may play non-necroptosis-associated roles in oocytes. pRIPK1, along with caspase 8, was shown to promote chromosome alignment during mitosis but was not shown to be localized near the spindle or kinetochore during this event [48]. Thus, what we observed in oocytes is unusual and has not been observed in other systems. Nec1 is an inhibitor o RIPK1 activity. Nec1 does not show cytotoxicity in mouse oocytes [25] or other cell types [49]. Since Nec1 treatment to block RIPK1 activity during vitrification reduced survival rate after warming, intact RIPK1 activity seems to be required for preserving oocyte quality. Further investigation is warranted to examine the necroptosis-dependent and -independent function of RIPK1 in oocyte biology and aging.

## Conclusions

Extending our previous findings that oocytes undergoing vitrification-warming show lipidome alterations, we conducted molecular analyses on lipid uptake- and necroptosis-associated factors. Our data provide information on the age-dependent change in *Cd36* expression and unique localization patterns of pMLKL and pRIPK1, components of necroptosis. Specific conditions that may activate this programmed cell death pathway in the context of cryopreservation of oocytes remains to be investigated.

## Data Availability

Data supporting findings are presented within the manuscript.

## Abbreviations

MII: Metaphase II
PMSG: Pregnant mare’s serum gonadotropin
hCG: human chorionic gonadotropin
qPCR: Real-time quantitative PCR
MLKL: Mixed lineage kinase-like protein
RIPK1: Receptor interacting protein kinase 1
MTOC: Microtubule organizing center
Nec1: Necrostatin 1
EG: Ethylene glycol
DMSO: Dimethyl sulfoxide
SRB1: Scavenger receptor class B type 1
SRB2: Scavenger receptor class B type 2
CD36: Cluster of differentiation 36
ESCRT: Endosomal sorting complex required for transport
Tsg101: Tumor susceptibility gene 101
Chmp4b: Charged multivesicular body protein 4B
ER: Endoplasmic reticulum
